# Genetics of Dentofacial and Orthodontic Abnormalities

**DOI:** 10.1055/s-0040-1722303

**Published:** 2021-02-01

**Authors:** Praveen Kumar Neela, Anjana Atteeri, Pavan Kumar Mamillapalli, Vasu Murthy Sesham, Sreekanth Keesara, Jaya Chandra, Udayini Monica, Vasavi Mohan

**Affiliations:** 1 Department of Orthodontics, Kamineni Institute of Dental Sciences, Narketpally, India; 2 Department of Genetics and Molecular Medicine, Vasavi Medical and Research Centre, Hyderabad, Telangana, India

**Keywords:** genetics, genes, polymorphism, dentofacial variations, malocclusion

## Abstract

The development of craniofacial complex and dental structures is a complex and delicate process guided by specific genetic mechanisms. Genetic and environmental factors can influence the execution of these mechanisms and result in abnormalities. An insight into the mechanisms and genes involved in the development of orofacial and dental structures has gradually gained by pedigree analysis of families and twin studies as well as experimental studies on vertebrate models. The development of novel treatment techniques depends on in-depth knowledge of the various molecular or cellular processes and genes involved in the development of the orofacial complex. This review article focuses on the role of genes in the development of nonsyndromic orofacial, dentofacial variations, malocclusions, excluding cleft lip palate, and the advancements in the field of molecular genetics and its application to obtain better treatment outcomes.

## Introduction

Genetics as an etiological factor plays a crucial role in the development of jaws, both maxilla, and mandible, dentition, and occlusion. In 1836, Frederick Kussel reported that malocclusion, both skeletal and dental, could be transmitted from one generation to another and also stated that chromosomal defects account for approximately 10% of all malocclusions. 1 The pioneering work of Gregor Johann Mendel initiated an interest in the field of genetics in the 19th century, and since then, genetics has been an essential part of the studies performed in various fields of biological and medical sciences. Since the 20th century, this branch of science has evolved through a series of era-based conceptual breakthroughs. Lack of sufficient understanding of principles of inheritance in the past led to a view regarding genetics and malocclusion remaining unclear. 2

Understanding the genetic factors contributing to the variation in dentofacial morphology associated with malocclusions is the key for proper diagnosis which, in turn, helps to develop novel treatment techniques. Advances in dentofacial phenotyping, which is the comprehensive characterization of hard- and soft-tissue variation in the craniofacial complex, together with the acquisition of large-scale genomic data have started to unravel genetic mechanisms underlying facial variation. Knowledge of the genetics of human malocclusion is limited even though results attained thus far are encouraging, with promising opportunities for future research. 3 This review article summarizes the role of genes in the development of nonsyndromic orthodontic, dentofacial variations, excluding clefts, malocclusions, and the advancements in the field of molecular genetics and its application to obtain better treatment outcomes.

## Genetic Influence on Skeletal Relationship of Jaws

Mandibular prognathism is caused by a deficiency of the maxillary growth, excessive mandibular growth, or a combination of both. Familial studies of mandibular prognathism are suggestive of heredity in the etiology of this condition. Various models have been suggested, such as autosomal dominant with incomplete penetrance, simple recessive, variable both in expressivity and penetrance with differences in different racial populations. 4 The familial nature of mandibular prognathism was first reported by Strohmayer as noted by Wolff et al. in their analysis of the pedigree of the Habsburg family. 5 The Habsburg jaw is seen in European royalty in which mandibular prognathism recurred over multiple generations. The genetic factors appear to be heterogeneous with monogenic influence (usually autosomal dominant with incomplete penetrance and variable expressivity) in some families and multifactorial (polygenic complex) influence in others. 6

Although various genetic linkage analyses and genome-wide association studies have identified many genes and loci associated with mandibular prognathism, the genes underlying the risk of mandibular prognathism in the general population remain ambiguous, leaving some impetus to search for new candidate genes. To date, genome-wide linkage analyses of mandibular prognathism have been performed among Japanese, Korean, Hispanic, and Chinese cohorts and several genetic loci have been reported to be associated with it, including 1p22.1, 1q32.2, 1p36,3q26.2, 4p16.1, 6q25, 11q22, 12q13.13, 12q23, 14q24.3–31.2, and 19p13.2. 7 8 9 10 11 12 13 Genome-wide linkage and association studies also found positive correlations for mandibular prognathism and genes, growth hormone receptor (GHR), 14 erythrocyte membrane protein band4.1 (EPB4), 15 synovial sarcoma X (SSX21), myosin1H (MYO1H), 16 collagen type II α1 (COL2A1), 17 fibroblast growth factor (FGF7), 18 transforming growth factor beta 3 (TGFB3), plexin A (PLXNA), latent transforming beta binding protein 2 (LTBP2), matrilin1 (MATN1), 9 dual specificity phosphatase 6 (DUSP6), 11 and a disintegrin and metalloproteinase with thrombospondin motifs 1 (ADAMTS1) 13 in various populations.

## Vertical Skeletal Jaw Abnormalities

Fontoura et al in their study suggested two candidate genes, PAX5, a transcription factor, and Rho GTPase activating protein 29 (ARHGAP29), which mediates the cyclical regulation of small GTP binding proteins such as RhoA, are associated with the vertical discrepancies, ranging from skeletal deep bite to open bite. 19 Manfredi et al in a more recent study on monozygotic twins, dizygotic twins, and same-sex siblings, assessed the inheritance traits of the orthodontic cephalometric parameters. They also suggested that the vertical parameters were more genetically controlled than the anteroposterior ones; heritability seemed to be expressed more anteriorly than posteriorly. The mandibular shape seemed to be determined more genetically than the mandibular size. 20 Savoye et al also reported similar findings and stated that the vertical proportions are highly under genetic control. 21

## Genetic Effects on Individual Tooth Variations

Genetic factors control the tooth size, morphology, number, position, and its inheritance, as stated in various twin studies. 22 23 24 HOX genes, which play a fundamental role in the oral and dental development, are known to show site-specific anteroposterior expression patterns. Molecular genetics of tooth morphogenesis, with the homeostatic Hox 7 and Hox 8 (presently MSX1 and MSX2) genes being responsible for stability in dental patterning, is confirmation of Butler's field theory. 25

A supernumerary tooth, which is most frequently seen in the premaxillary region with a greater prevalence for males, also appears to be genetically determined. Niswander and Sujaku 26 in 1963 analyzed data from family studies, and they suggested that, like hypodontia, the genetics of less prevalent condition of supernumerary teeth is under control of several genes in different loci and may be associated with an autosomal recessive gene with lesser penetrance in females. This was later supported by Galas and Garcia in 1999. 27 While an autosomal dominant inheritance with incomplete penetrance has been suggested, the increased incidence in males suggests the possibility of sex-linked heredity, as stated by Bruning et al. Although this inheritance does not follow a simple Mendelian pattern, these are more commonly present in parents and siblings of patients who present with this condition. Evidence from twins with supernumerary teeth also supports this theory. 28

Dental agenesis, which is the most common developmental anomaly seen in humans, is genetically and phenotypically a heterogeneous condition. Based on the current knowledge of genes and the factors involved in the tooth development and morphogenesis, it is assumed that different phenotypic forms are caused by different genes involving different interacting molecular pathways, providing an explanation not only for the wide variety in agenesis patterns but also for associations of dental agenesis with other oral anomalies. More than 200 genes have so far been identified, which are expressed during tooth development, and mutations in several of these genes are known to cause arrested tooth development in mice. 29

Population studies have shown that tooth agenesis can be manifested as an isolated trait or part of a syndrome. Isolated forms may be either sporadic or familial. Familial tooth agenesis can be the result of a single dominant gene defect or recessive or X-linked. Third molar agenesis cannot be explained in most of the cases with a simple model of autosomal dominant transmission. Besides, a polygenic mode of inheritance has also been reported in the literature. Grahnen 30 stated that tooth agenesis is typically transmitted as an autosomal dominant trait with incomplete penetrance and variable expressivity. Twin studies have been widely used to show the importance of the genetic component involved during tooth development to control both tooth size and form. There are numerous case reports, suggesting concordance for tooth agenesis in monozygotic twins, and case reports where variation in the expressivity is observed. 31

Numerous mutations in transcription factor and growth factor-related genes involved in dental development have been shown to play a role in human dental agenesis, including paired box 9 (PAX9), a transcription factor, and muscle segment homeobox 1 (MSX1). MSX1 gene mutations can lead to hypodontia or oligodontia as well as variations in the downstream signaling gene bone morphogenetic protein 4 (BMP4). In humans, a point mutation in MSX1 homeobox results in agenesis of second premolars and third molars in affected individuals. 25

Mutations in PAX9 typically show a nonsyndromic autosomal dominant mode of inheritance for oligodontia, with variable expressivity within families. The characteristic pattern of dental agenesis caused by PAX9 mutations primarily affects molars in both dental arches and second premolars most often in the maxillary arch than the mandibular arch, occasionally presenting with missing or peg-shaped mandibular central incisors and maxillary lateral incisors. Agenesis of maxillary first premolars or canines can occur with a low frequency among PAX9 mutations. In contrast, the PAX9 Ala240Pro mutation may be unique, in that it leads uniquely to third molar agenesis with or without affected incisors. Mutations in the axis inhibitor 2 gene (AXIN2) have also been linked to oligodontia, often exhibiting a similar pattern of affected teeth as PAX9 mutations. 32 From this, it is clear that the functions of PAX9 and MSX1 are essential for the establishment of the odontogenic potential of the mesenchyme through the maintenance of mesenchymal Bmp4 expression. However, the relationship between these three genes on the molecular level remains unknown.

Primary failure of eruption (PFE), which was described initially by Profitt and Vig, 33 is characterized by nonsyndromic eruption failure of permanent teeth in the absence of mechanical obstruction. Many studies have stated the heritable basis of this dental phenotype, and recently, mutations in parathyroid hormone receptor 1 (PTH1R) have been identified. It functions in signaling in mesenchymal progenitors, alveolar bone formation, and periodontal ligament development during eruption physiology. The recent report of PTH1R mutations associated with primary failure of eruption makes this a high-priority candidate gene for confirming the diagnosis of a nonsyndromic PFE phenotype. 34

Crowding of teeth is a complex dental anomaly that affects esthetics and quality of life. Crowding is usually caused by insufficient arch space that cannot accommodate all erupting permanent teeth. Genetics is suggested to contribute to the etiology of crowding. A study conducted by Ting et al suggested a significant association for the genes ectodysplasin A (EDA) and X-linked ectodermal dysplasia receptor (XEDAR), which are important in the signaling pathway that plays a role in the development of dental crowding among the Hong Kong Chinese population. Since this association study was done in the Hong Kong Chinese population, the results might not apply to other ethnic groups. Further replication studies in other ethnic groups with a larger sample size are vital for confirmation of these findings. 35

A genetic tendency for ectopic maxillary canines has also been reported in various association studies. 36 Peck et al concluded that palatally ectopic canines, as an inherited trait, is one of the anomalies in a complex of genetically related dental disturbances, often occurring in combination with missing teeth, tooth size reduction, supernumerary teeth, and other ectopically positioned teeth. 37 Previous studies have also shown an association between ectopic maxillary canines and class II division 2 malocclusion, a genetically inherited trait. 38

Genetic variation showing a significant effect on arch width and length was confirmed in various studies on monozygotic and dizygotic twins. A genetic contribution to arch shape was found by Richards et al after comparing the intraclass correlations between monozygotic and dizygotic South Australian twins. 39

A study was conducted by Corruccini et al on the occlusal characteristics in 32 pairs of monozygotic twins and 28 pairs of dizygotic twins using dental stone casts. They studied arch shape, size, and symmetry, overjet, overbite, posterior crossbite, buccal segment relation, rotation, and displacements. They concluded that arch size variation, tooth displacement, and crossbite showed significant genetic variance and also found an increased environmental component of variance in occlusion. 40 A study on north-west Indian twins revealed significant genetic variance for dental arch and palate dimensions, but environmental influences seemed important for occlusal traits. 41

## Effects of Genetics on Inheritance of Malocclusion

Malocclusion is a significant deviation from an ideal or normal occlusion. 38 Malocclusion can either be skeletal or dental, involving discrepancies in the jaw size, tooth size, and shape, crowding, or spacing. It is a manifestation of both genetic factors and environmental influences during the development of the craniofacial complex. However, it might be difficult to differentiate whether the malocclusions are determined by the genetic code or environmental factors, or a combination of both. 42 The concepts and principles of molecular genetics have become significant components in the understanding of the genesis of variations in the growth, development, and form of the entire craniofacial complex.

Genetic factors playing a predominant role in the etiology of malocclusion is backed by population studies, especially family and twin studies. 43 Familial aggregation studies suggested that an autosomal dominant model with incomplete penetrance has the most significant validity for Class III pedigrees, including the royal Habsburg family 5 and others from middle Eastern, 44 South American, 16 45 and Eastern European descent. In contrast, polygenic inheritance and autosomal dominance models, with incomplete penetrance and variable expressivity, have been suggested for Class II division 1 and 2, respectively. 46 Lauweryns in 1993 reported that 40% of the dental and skeletal variations that lead to malocclusion could be attributed to genetic factors. 47

Extensive cephalometric studies by Harris suggested the concept of polygenic inheritance for Class II division 1 malocclusion, showing that the craniofacial skeletal patterns of children with class II malocclusions are heritable and that there is a high resemblance to the skeletal patterns in their siblings with normal occlusion. 48

Familial occurrence of Class II division 2 has been documented in several published reports including twin and triplet studies (Kloeppel; Markovic) and family pedigrees from Korkhaus, Rubbrecht, Trauner, 49 and Peck et al. 50 Twin studies showed that the identical twins demonstrated 100% concordance for Class II division 2 malocclusion, indicating a strong genetic influence in the development of Class II division 2 deep bite malocclusions. 51 Markovic's clinical and cephalometric study of intra- and interpair comparisons of 114 Class II division 2 malocclusions, 48 twin pairs, and six sets of triplets showed complete penetrance and variable expressivity of autosomal dominant genetic impression. 52 The controversy regarding the etiology of the Class II Division 2 malocclusion arises from a failure to appreciate the synergistic effects of genetics and the environment on facial morphology. Ballard, Houston, Mills, 53 and others considered that a high lip line and a particular lip morphology and behavior were the main etiological factors. Graber, Hotz, Meskov and Markovic 54 stressed the predominant role of genetic factors in the etiology of Class II division 2 malocclusions.

Nakashima et al. 55 conducted a study to assess the role of heredity in the development of Angle's Class II and Class Ill malocclusions, and their results showed that: 1) The parents of Class II patients had a convex profile with a distocclusion type of denture pattern, while the parents of Class III patients had a concave profile with a mesiocclusion type of denture pattern, suggesting both Class II and Class III malocclusions have a genetic basis. These correlations between parent and offspring were stronger for the skeletal measurements in both classes. 2) The correlation coefficients in the parent-offspring data were in good agreement with the expected level under the polygenic model of inheritance. 3) Significant differences between Class II and III patients for four variables (upper incisor to NA angle, gonial angle, Ar-Go, and upper incisor to nasal floor angle) were considered to be related to environmental factors. 55

## Genetic Effects on External Apical Root Resorption

Evidence from previous studies suggest that genetic factors play a significant role in the development of root resorption. 56 Al-Qawasmi and colleagues in 2003 performed a family study to assess the potential effect of single nucleotide polymorphisms (SNPs) in two closely-located proinflammatory candidate genes (IL-1A and IL-B) on root resorption and found that patients who were homozygous for IL-1B allele 1 have a 5.6 fold (95% CI 1.9–21.2) increased risk of apical root resorption (ARR) compared with those who were not homozygous for the IL-1B allele. 57 Another candidate locus identified to be associated with the development of ARR is located on chromosome 18 and showed evidence for linkage between a microsatellite marker D18S64, which lies close to the candidate gene TNF receptor superfamily member 11a (TNFRSF11A) and root resorption trait. The TNFRSF11A gene encodes the receptor activator of nuclear factor-kappa B (RANK), an essential signaling molecule in osteoclast formation and activation as a potential mechanism in the pathogenesis of root resorption. 58

## Genetic Implications on Orthodontic Tooth Movement

Multiple molecular pathways that influence orthodontic tooth movement (OTM) are identified to date. Two of the pathways that influence both orthodontic tooth movement and external apical root resorption include the ATP/P2XR7/IL-1B inflammatory signaling pathway and the RANKL/RANK/OPG bone modelling and remodeling pathway. However, even with this knowledge of key pathways influencing OTM, few studies have focused on determining how actual variations in nonsyndromic genetic factors correlate with the actual clinical outcomes observed during OTM in humans. 59 Studies have been done with genetic variation markers based on the part of the ATP/P2RX7/ IL-1B pathway, the genes for IL-1β and another related cytokine interleukin 1 α IL-1α (IL1B and IL1A, respectively), and the gene (IL1RN) for another molecular pathway (IL-1 receptor antagonist, IL-RA) that helps to regulate their biological activity. 60 Of these two forms, interleukin 1 β-IL-1β is the most potent for bone resorption and inhibition of bone formation. OTM requires a balance between IL-1β and IL-1RA synthesis for the bone modelling and remodeling processes involved. 61

## Conclusion

The knowledge of the role of genetics is essential for the orthodontist which helps to understand why a patient has a particular occlusion, because malocclusion is a manifestation of genetic and environmental interaction on the development of the orofacial complex. Awareness regarding the genetic expression of the dentofacial maldevelopment is an essential aid in the correction of malocclusion, as it helps to segregate the inherited malocclusions from those due to the effect of environmental factors and thereby helps to diagnose, treat, and possibly even prevent a malocclusion from occurring in the next generation.

There has been immense progress in the field of genetically supported orthodontics to date. Although it is very challenging to reveal the genetic component of most malocclusions and dental anomalies because of its polygenic nature, data developed and provided by the human genome project have made it feasible to map inherited conditions related to the dentofacial development. However, further genetic studies are required to determine all the specific genes leading to a particular skeletal variability. Genome-wide association studies are necessary to evaluate further as well as provide a database for evidence-based practice.
